# Clinical and Imaging Patterns of Neurological Involvement in Intravascular Large B-Cell Lymphoma: A Single-Center Retrospective Study †

**DOI:** 10.3390/diagnostics16203405

**Published:** 2026-10-20

**Authors:** Yiao Di, Chang Geng, Siyuan Fan, Chao Chen, Min Lang, Wei Zhang, Daobin Zhou, Yan Zhang

**Affiliations:** 1 Department of Hematology, Peking Union Medical College Hospital, Chinese Academy of Medical Sciences & Peking Union Medical College, Beijing 100730, China; 2 Department of Neurology, Peking Union Medical College Hospital, Chinese Academy of Medical Sciences & Peking Union Medical College, Beijing 100730, China

**Keywords:** intravascular large B-cell lymphoma, neurological involvement, neuroimaging, clinicoradiologic patterns, myelopathy, radiculoneuropathy

## Abstract

**Objective:** Neurological involvement in intravascular large B-cell lymphoma (IVLBCL) is heterogeneous, prompting us to characterize its diverse manifestations through an integrated clinical and neuroimaging framework. **Methods:** This single-center retrospective study included 55 patients with pathologically confirmed IVLBCL diagnosed at Peking Union Medical College Hospital between January 2015 and June 2023. Pretreatment neurological involvement was identified in 29 patients based on attributable clinical and/or imaging findings after exclusion of more plausible alternative causes. Patterns were assigned primarily by clinical manifestations, supported by available imaging. **Results:** Six potentially overlapping clinicoradiologic patterns were identified. **The stroke-like pattern (*****n*** **= 5)** comprised acute focal deficits with corresponding infarct-like or ischemic lesions. **The encephalopathy pattern (*****n*** **= 11)** included cognitive decline, behavioral changes, impaired consciousness, and/or seizures, with or without infarct-like lesions or white matter hyperintensities. Three patients developed encephalopathic features following stroke-like presentations, including one with concomitant myelopathy. **The mass-like pattern (*****n*** **= 1)** presented with headache and vomiting associated with enhancing lesions and surrounding edema. **Myelopathy (*****n*** **= 3)** was characterized by lower-limb weakness with spinal cord-localizing signs, whereas **radiculoneuropathy (*****n*** **= 5)** manifested as bilateral lumbosacral nerve-root involvement with weakness, sensory abnormalities, and reduced reflexes. Corresponding MRI abnormalities were absent in two patients with encephalopathy and one with myelopathy. **The asymptomatic pattern (*****n*** **= 8)** comprised patients with imaging abnormalities but no neurological symptoms or signs. A total of 19 of 22 patients evaluable for treatment response achieved complete remission. **Conclusions:** Neurological involvement in IVLBCL comprises overlapping and evolving clinicoradiologic presentations, with variable correspondence between symptoms and imaging findings. This descriptive framework may help clinicians recognize its diverse manifestations and consider IVLBCL when the clinical course or imaging findings remain insufficiently explained.

## 1. Introduction

Intravascular large B-cell lymphoma (IVLBCL) is a rare, aggressive lymphoma characterized by the almost exclusive growth of neoplastic cells within the vascular lumen, typically with rapid progression and highly heterogeneous systemic manifestations [1,2,3,4]. When the nervous system is involved, IVLBCL may mimic more common neurological disorders, including acute ischemic stroke, encephalopathy, and inflammatory or demyelinating diseases [4,5,6]. Historically, postmortem diagnosis was particularly common in cases with central nervous system involvement [7], underscoring the diagnostic difficulty of neurologically predominant IVLBCL.

Previous studies have outlined a broad and heterogeneous spectrum of neurological involvement in IVLBCL. Cerebral MRI studies have described overlapping infarct-like, white matter, meningeal, mass-like, and pontine patterns [6,8], while clinical investigations have highlighted recurrent or fluctuating focal neurological episodes and rapidly progressive cognitive decline [9]. Beyond cerebral involvement, IVLBCL-associated longitudinally extensive or conus-predominant myelopathy, as well as cauda equina and radicular syndromes, have also been documented [10,11,12,13]. Despite these advances, clinical syndromes and imaging abnormalities have often been described separately, and their correspondence across different neurological compartments remains incompletely defined. We therefore integrated clinical and imaging findings to characterize overlapping patterns of cerebral, spinal cord, and nerve-root involvement and to provide a descriptive framework that may facilitate clinical recognition.

## 2. Methods

### 2.1. Study Design, Patient Enrollment and Assessment of Neurological Involvement

This single-center retrospective study included 55 consecutive patients with pathologically confirmed IVLBCL who were diagnosed at Peking Union Medical College Hospital between January 2015 and June 2023. Clinical, laboratory, imaging, treatment, and follow-up data were retrospectively collected from electronic medical records.

In patients with pathologically confirmed IVLBCL, neurological involvement was assessed using clinical and imaging findings documented before the initiation of lymphoma-directed treatment, including neurological symptoms or signs and/or abnormalities involving the brain, pituitary gland, spinal cord, or nerve roots on MRI, CT, or ^18^F-FDG PET/CT. Patients with imaging abnormalities at these sites but no neurological symptoms or signs were also assessed for possible neurological involvement. Further investigations, including laboratory testing, cerebrospinal fluid analysis, cerebrovascular evaluation, and electrophysiological studies, were performed to investigate alternative causes, such as cerebrovascular disease unrelated to IVLBCL, autoimmune encephalitis, demyelinating or infectious diseases, paraneoplastic neurological syndromes, metabolic disorders, and other nervous system neoplasms. Neurological involvement was attributed to IVLBCL when the clinical course and available investigation results supported this interpretation and no alternative diagnosis provided a more plausible explanation. Histopathological confirmation of nervous system involvement was not a prerequisite.

**Figure 1:**
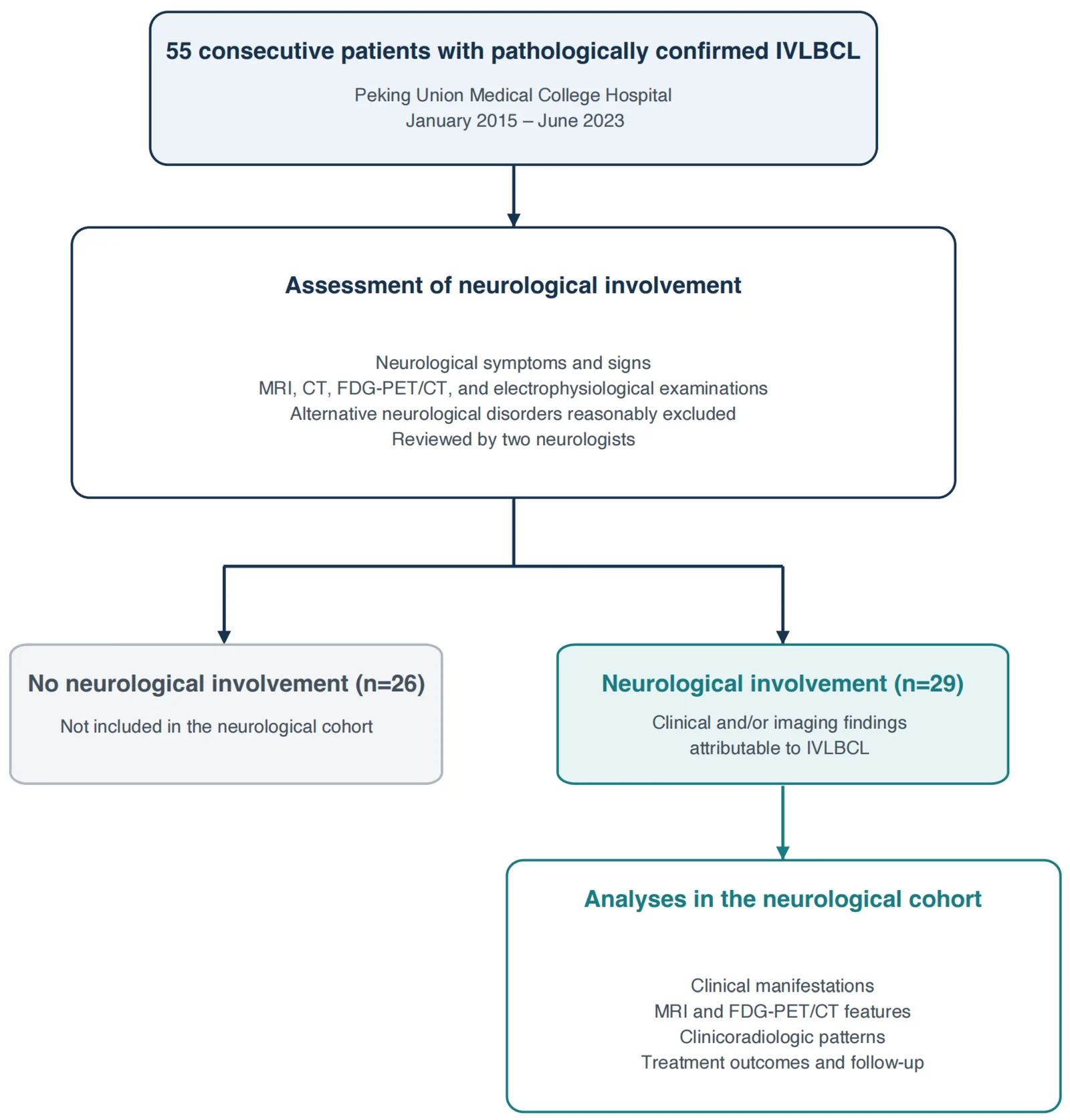
Patient enrollment and study workflow.

All available clinical, laboratory, and neuroimaging data were independently reviewed by two neurologists, and disagreements were resolved by consensus. Following this assessment, 29 patients were determined to have neurological involvement and constituted the neurological cohort for subsequent analyses (Figure 1).

### 2.2. Operational Definitions of Clinicoradiologic Patterns

Six descriptive clinicoradiologic patterns were defined through retrospective review, primarily according to clinical syndromes and neurological localization, with imaging and electrophysiological findings providing supporting evidence.

**The stroke-like** **pattern** comprised acute focal neurological deficits suggestive of cerebral ischemia, including hemiparesis, hemisensory disturbance, facial weakness, or transient aphasia. Corresponding ischemic or infarct-like lesions supported classification but were not mandatory for transient-ischemic-attack (TIA) -like episodes. Infarct-like imaging findings without an acute focal syndrome were insufficient for assignment to this pattern.

**The encephalopathy pattern** comprised cognitive decline, psychiatric or behavioral disturbances, otherwise unexplained impairment of consciousness, and/or seizures, with or without associated white matter signal abnormalities.

**The mass-like pattern** required an intraparenchymal mass-like lesion on imaging with associated clinical manifestations, such as focal neurological dysfunction, seizures, or symptoms suggestive of raised intracranial pressure.

**The myelopathy pattern** comprised motor, sensory, and/or autonomic dysfunction localizing to the spinal cord, supported by findings such as pyramidal signs or a sensory level. In patients with incomplete or atypical clinical findings, an anatomically concordant spinal cord lesion on MRI provided additional evidence for classification. Cases with suggestive symptoms but insufficient localizing evidence were recorded separately as suspected myelopathy.

**The radiculoneuropathy pattern** comprised weakness and/or sensory disturbances in a nerve-root or peripheral nerve distribution, often accompanied by reduced muscle tone or tendon reflexes. Sphincter dysfunction could accompany lumbosacral root involvement. Concordant neurogenic abnormalities on electrophysiological testing or nerve-root thickening and/or enhancement on MRI supported classification when available.

**The asymptomatic pattern** comprised imaging-detected involvement of the brain, pituitary gland, spinal cord, or nerve roots without neurological symptoms or signs. Isolated pituitary involvement with endocrine abnormalities was considered neurologically asymptomatic.

These patterns were descriptive rather than validated diagnostic criteria. Patients with clinical evidence of more than one symptomatic pattern were assigned to each applicable category.

### 2.3. Treatment and Follow-Up

Patients received predominantly R-CHOP-based immunochemotherapy. Some patients additionally received methotrexate and/or a Bruton tyrosine kinase (BTK) inhibitor, such as zanubrutinib. The planned treatment course generally consisted of eight cycles, with an interim assessment after four cycles and an end-of-treatment assessment after eight cycles. Treatment response was assessed according to the Lugano classification, with PET/CT findings interpreted using the Deauville five-point scale when available [14]. Follow-up data were collected from medical records, outpatient visits, and telephone interviews to document disease progression or relapse and survival status.

### 2.4. Statistical Analysis

Descriptive analyses were performed using IBM SPSS Statistics, version 27.0. Continuous variables were summarized as medians with ranges or interquartile ranges, as appropriate, and categorical variables as counts and percentages. As not all patients underwent every investigation, the number of patients tested was reported for each investigation, and percentages were calculated using the corresponding denominator. The overall response rate was defined as the proportion of evaluable patients achieving complete or partial remission.

### 2.5. Use of Generative AI

Generative AI (GPT-6 Astra, OpenAI) was used to assist with literature searching and language editing. The study conception, clinical adjudication, interpretation of findings, and final manuscript decisions were undertaken by the authors. All AI-assisted content was reviewed and verified by the authors.

## 3. Results

### 3.1. Cohort Characteristics

A total of 29 patients with pathologically confirmed IVLBCL and neurological involvement were included in this study (Table 1). The median age was 57 years (IQR, 52–64), and 18 patients (62.1%) were male. All patients had Ann Arbor stage IV disease, and the median International Prognostic Index (IPI) score was 4.

Twenty-one patients (72.4%) had neurological symptoms or signs. Focal neurological deficits occurred in five patients (17.2%), including unilateral limb weakness, hemisensory disturbance, facial weakness, and speech disturbance. Encephalopathic manifestations included cognitive impairment or slowed responses in 10 patients (34.5%), seizures in six (20.7%), and impaired consciousness in seven (24.1%). One patient (3.4%) presented with headache and vomiting associated with intracranial mass-like lesions. Manifestations relevant to spinal cord or nerve-root/peripheral nerve involvement included bladder and/or bowel dysfunction in 12 patients (41.4%) and bilateral lower-limb weakness in nine (31.0%); a sensory level was documented in three (10.3%). These manifestations were not mutually exclusive. The remaining eight patients (27.6%) had no neurological symptoms or signs. The predominant systemic manifestations were cytopenia (79.3%), fever (72.4%), and hepatosplenomegaly (41.4%).

Laboratory investigations showed a median serum LDH level of 896 U/L among all 29 patients and a median serum IL-10 level of 323 pg/mL among the 25 patients tested. All patients underwent lumbar puncture. CSF leukocyte counts were generally normal (median, 2 × 10^6^/L; IQR, 0–4), with mildly elevated protein levels (median, 0.72 g/L; IQR, 0.39–1.17). No malignant cells were detected on CSF cytology. The median CSF IL-10 level was 6 pg/mL (IQR, 5–25) among the 22 patients tested. Skin was the most frequent site yielding a positive biopsy result (17, 58.6%), whereas bone marrow biopsy was positive in three patients (10.3%). IVLBCL was diagnosed by brain biopsy in only three patients (10.3%).

### 3.2. Radiological Findings

All 29 patients underwent at least one form of neuroimaging evaluation, including brain, spinal cord, or lumbosacral nerve-root MRI, CT, or FDG-PET/CT. Brain MRI was available for review in 23 patients, of whom 15 had intracranial lesions, with multiple lesions (13, 56.5%) outnumbering those with a single lesion (2, 8.7%) (Table 2). The subcortical white matter was the most frequently involved intracranial region (12/23, 52.2%), followed by the brainstem (7/23, 30.4%).

**Figure 2:**
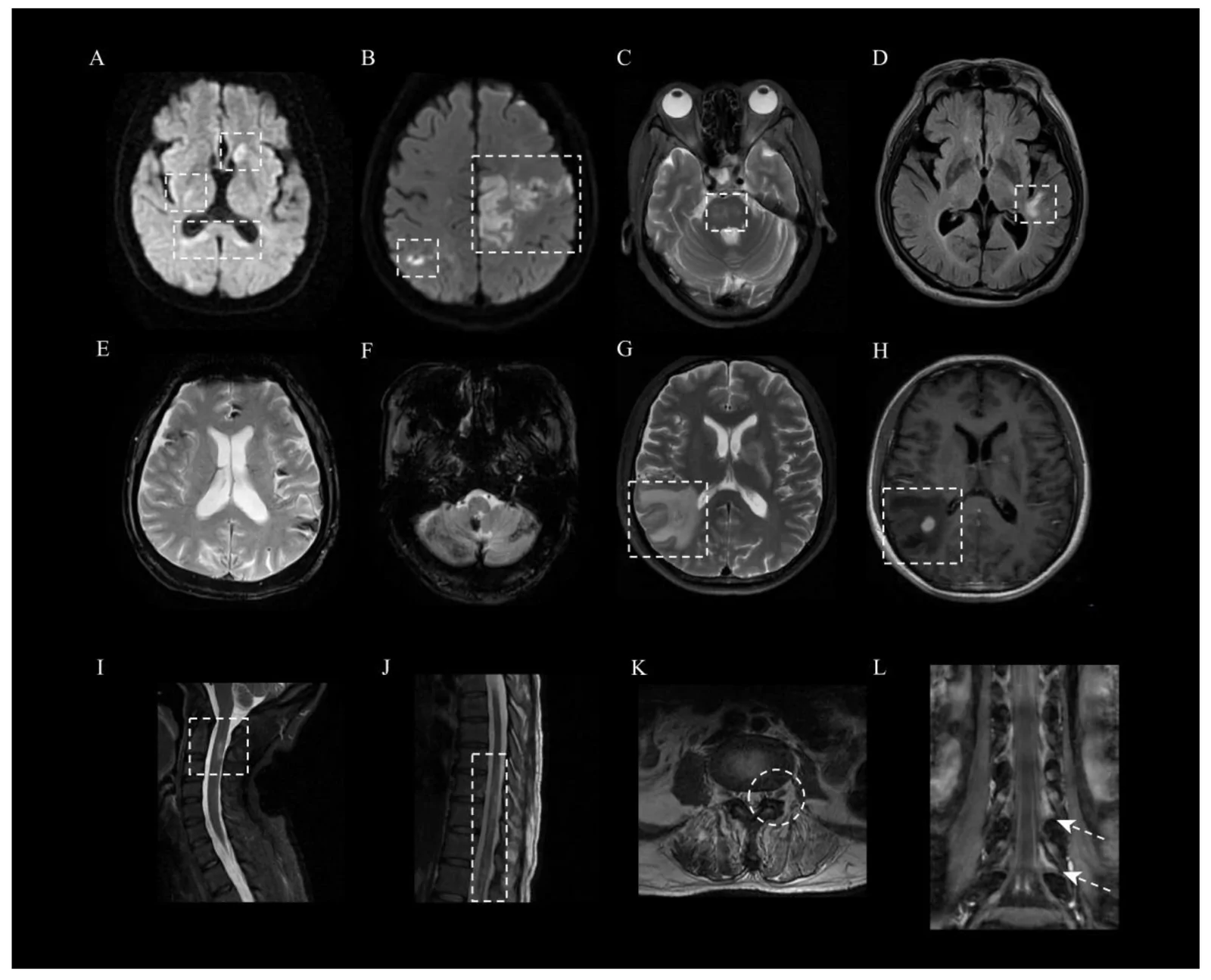
Abnormal neuroimaging findings. (**A**,**B**) Multifocal diffusion-restricted lesions on DWI involving multiple vascular territories. (**C**,**D**) White matter hyperintensities involving both pons and subcortical regions on T2-weighted and FLAIR imaging. (**E**,**F**) Multifocal hemosiderin deposition on T2*-weighted imaging. (**G**,**H**) Mass-like lesion with surrounding edema and contrast enhancement. (**I**,**J**) Lesions involving the cervical and thoracic spinal cord. (**K**,**L**) Thickening in T2-weighted imaging and contrast enhancement in T1-weighted imaging of the lumbosacral nerve roots.

Infarct-like or ischemic lesions were identified in nine patients (39.1%). Of these, seven (30.4%) had multifocal infarct-like lesions, typically showing diffusion restriction across multiple vascular territories (Figure 2A,B), and two (8.7%) had ischemic changes without definite acute infarction. White matter hyperintensities were observed in 10 patients (43.5%), with representative T2-weighted and FLAIR abnormalities involving the pons and subcortical white matter (Figure 2C,D). Hemorrhagic lesions were identified in three patients (13.0%), appearing as multifocal susceptibility hypointensities consistent with hemosiderin deposition (Figure 2E,F). Multiple enhancing mass-like lesions with surrounding edema were uncommon, occurring in one patient (4.3%) (Figure 2G,H). Spinal and nerve-root MRI were performed in patients with suspected spinal cord or peripheral nervous system involvement. Spinal cord T2 hyperintensities were identified in two patients, involving the cervical and thoracic cord (Figure 2I,J). Lumbosacral nerve-root thickening was observed in four patients, with associated contrast enhancement in two (Figure 2K,L).

^18^F-FDG-PET/CT was available for 22 patients. Hypermetabolism involving neural structures or the pituitary gland was identified in 11 patients (50.0%), involving cerebral or meningeal regions in four (18.2%), nerve roots in one (4.5%), and the pituitary gland in seven (31.8%), with overlapping sites. Among the 16 patients with both brain MRI and PET/CT available, hypermetabolism corresponded to T2-hyperintense lesions in two (12.5%) and enhancing mass-like lesions in one (6.3%), whereas hypometabolism corresponded to ischemic lesions in one (6.3%). In six patients (37.5%), brain MRI lesions showed no corresponding abnormal FDG uptake. Four patients (25.0%) had neither brain MRI lesions nor abnormal FDG uptake in neural structures or the pituitary gland. Two patients (12.5%) had no brain MRI lesions but showed nerve-root or pituitary hypermetabolism, respectively. Among patients without evaluable brain MRI, three showed pituitary hypermetabolism on PET/CT without abnormal uptake elsewhere in the brain. One presented with seizures and altered consciousness, while the other two had no neurological symptoms or signs. All three patients with documented pituitary-related endocrine abnormalities showed pituitary hypermetabolism on FDG-PET/CT.

### 3.3. Clinicoradiologic Patterns of Nervous System Involvement

**Figure 3:**
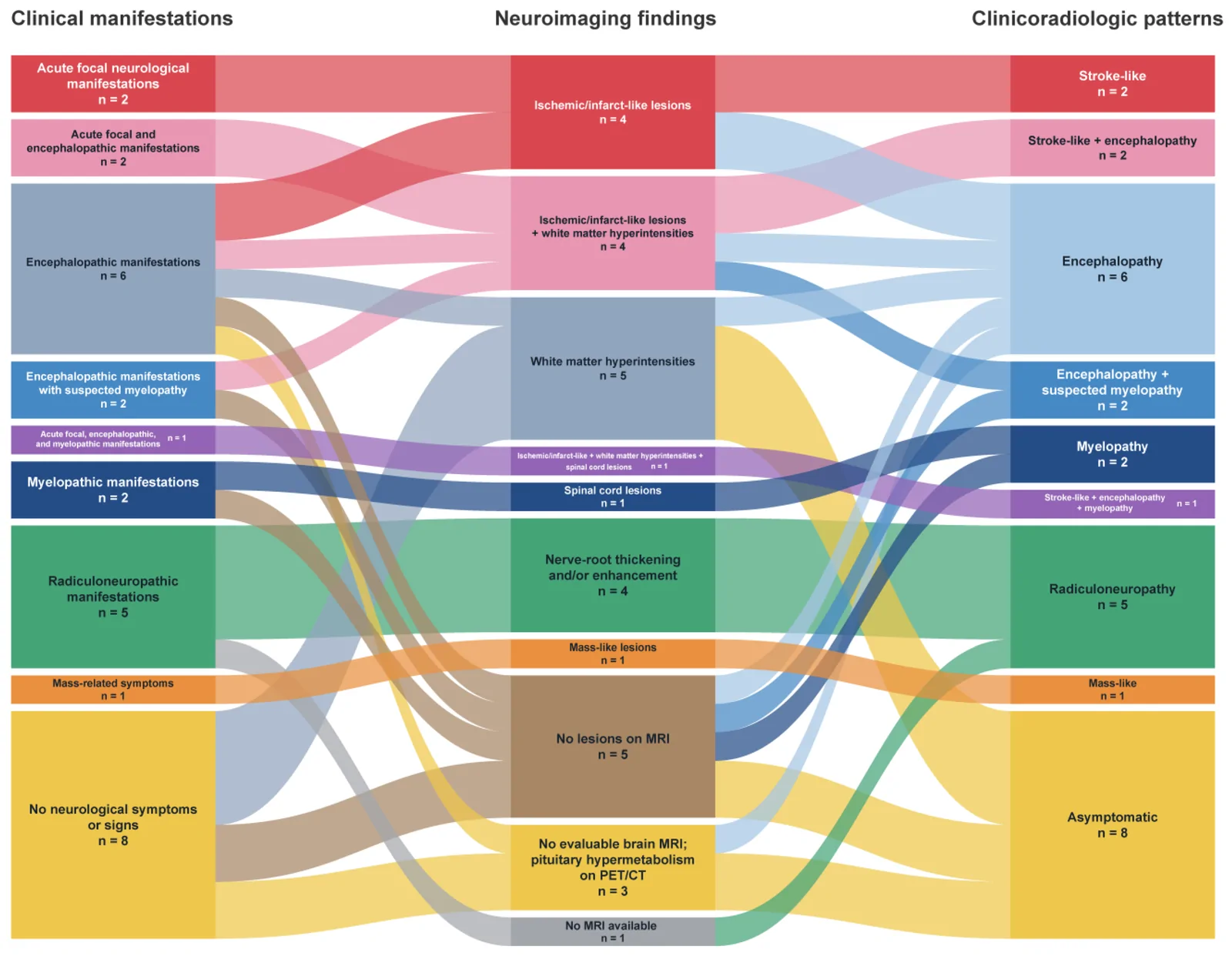
Sankey diagram illustrating clinicoradiologic patterns in IVLBCL with neurological involvement. Individual patients could exhibit multiple clinical and neuroimaging features, resulting in overlapping clinicoradiologic patterns. Each patient is counted once per column, with overlapping features or patterns represented by nodes. “No lesions on MRI” denotes no relevant lesions on available brain, spinal cord, or nerve-root MRI.

Based on the definitions provided in Section 2, patients were assigned to six potentially overlapping clinicoradiologic patterns primarily according to their clinical manifestations, supported by neuroimaging findings: stroke-like, encephalopathy, myelopathy, radiculoneuropathy, mass-like, and asymptomatic patterns (Figure 3).

Stroke-like and encephalopathy patterns were identified in 5 and 11 patients, respectively. All patients with stroke-like presentations had anatomically corresponding infarct-like lesions or ischemic changes on brain MRI. Some experienced recurrent focal neurological episodes and received secondary stroke prevention for presumed cerebrovascular disease, followed by further neurological episodes and the emergence of systemic manifestations. One patient had predominantly systemic manifestations and experienced a transient episode of hemiparesis and aphasia during the disease course that resolved spontaneously within 6 h. Brain MRI showed multifocal ischemic changes, and the episode was considered a transient ischemic attack (TIA). Among patients with encephalopathy, two had no brain MRI lesions, while one had no evaluable brain MRI and no abnormal cerebral parenchymal FDG uptake, despite pituitary hypermetabolism. Three patients exhibited both stroke-like and encephalopathic features. In all three, focal neurological symptoms preceded cognitive decline, behavioral changes, or reduced responsiveness. One subsequently experienced another acute episode of unilateral weakness and numbness after encephalopathic features had developed. By contrast, four patients had infarct-like or ischemic lesions on MRI but encephalopathic manifestations without an acute focal neurological syndrome, and were therefore assigned to the encephalopathy pattern.

The myelopathy pattern was considered in three patients, including two with spinal cord lesions on MRI, one of whom also exhibited prominent stroke-like and encephalopathic patterns. The remaining patient had characteristic clinical findings localizing to the spinal cord despite a negative spinal MRI. Two additional patients had encephalopathy with suspected myelopathy: lower-limb pain on touch, progressive weakness, and urinary difficulty preceded cognitive decline and disorientation. Neither underwent spinal MRI. Brain MRI revealed definite cerebral lesions in one, whereas brain MRI and FDG-PET/CT showed no relevant abnormalities in the other. Spinal cord involvement therefore remained clinically suspected in these two patients.

The radiculoneuropathy pattern was identified in five patients, predominantly presenting with bilateral lower-limb weakness, sensory disturbances, and reduced tendon reflexes, consistent with bilateral lumbosacral nerve-root involvement. MRI demonstrated lumbosacral nerve-root thickening in four patients, with associated enhancement in two. Electromyography showed neurogenic changes in two patients. In one of these patients, neither brain nor lumbosacral nerve-root MRI was available; peripheral nervous system involvement was supported by clinical and electrophysiological findings.

The mass-like pattern was uncommon and was identified in only one patient, who presented with headache and vomiting. The imaging findings initially raised suspicion of brain metastases; however, IVLBCL was subsequently diagnosed through an abdominal wall skin biopsy.

The asymptomatic pattern was identified in eight patients (27.6%). Four had white matter hyperintensities on brain MRI, including two with solitary lesions and two with multiple lesions. The remaining four showed pituitary hypermetabolism on FDG-PET/CT; brain MRI was negative in two and unavailable for review in the other two. Two of these four patients had pituitary-related endocrine abnormalities.

### 3.4. Treatment and Outcomes

A total of 24 patients received rituximab-based chemotherapy following pathologic confirmation of IVLBCL, including 16 patients treated with R-CHOP regimens combined with a bruton tyrosine Kinase inhibitor (BTKi) such as zanubrutinib. Among 22 evaluable patients, 19 achieved complete remission (CR), two achieved partial remission (PR), and one had stable disease (SD), yielding an overall response rate of 95.5%.

During a median follow-up of 30 months (IQR 9–40), four patients died, including three patients who did not receive chemotherapy. Despite favorable hematologic responses, residual neurologic sequelae persisted in a substantial proportion of patients with nervous system involvement.

## 4. Discussion

In this single-center retrospective study from China, neurological involvement was identified in 29 of 55 patients with IVLBCL (52.7%), encompassing the brain, pituitary gland, spinal cord, and peripheral nervous system. By integrating clinical manifestations with neuroimaging findings, we described six potentially overlapping clinicoradiologic patterns: stroke-like, encephalopathy, myelopathy, radiculoneuropathy, mass-like, and asymptomatic. We characterized their coexistence and sequential emergence within individual patients, as well as the correspondence and discrepancies between clinical and imaging findings. This framework aims to facilitate recognition of this broad spectrum of neurological involvement and help clinicians consider IVLBCL when the clinical course or imaging findings are not adequately explained by the initial diagnosis.

Multifocal infarct-like lesions were an important manifestation of cerebral involvement in our cohort. Three patients developed cognitive decline, behavioral changes, or reduced responsiveness after focal neurological episodes, and one subsequently experienced another acute focal episode. This course is consistent with the coexistence and temporal evolution of stroke-like episodes and cognitive deterioration reported by Berthet et al. and with recent descriptions of recurrent stroke preceding the diagnosis of IVLBCL [9,15]. Progressive cerebral dysfunction may reflect cumulative ischemic and white matter injury associated with ongoing microvascular involvement. Previous pathological studies have demonstrated intravascular tumor cells accompanied by vascular occlusion, infarction, necrosis, congestion, and demyelination, providing a plausible basis for these heterogeneous manifestations [6].

However, multiple cerebral infarcts involving different vascular territories and primary CNS vasculitis can also present with both focal deficits and encephalopathic features, with MRI findings that overlap with those of IVLBCL [16,17]. When systemic features such as fever or hepatosplenomegaly are absent at presentation, this resemblance may lead to treatment directed at presumed ischemic stroke or vasculitis, potentially delaying recognition of the underlying lymphoma. Elevated LDH, cytopenia, or otherwise unexplained inflammatory abnormalities may provide additional clues prompting investigation for IVLBCL [9]. Skin biopsy demonstrated IVLBCL in 17 of our 29 patients, highlighting the diagnostic contribution of accessible extracranial tissue. When suspicion remains despite the absence of visible skin lesions, random skin biopsy offers a relatively less invasive route to tissue diagnosis, although a negative result does not exclude IVLBCL [18].

The patient with a mass-like presentation further illustrates this diagnostic approach. Small enhancing lesions with extensive surrounding edema initially suggested brain metastases, but an abdominal wall skin biopsy established IVLBCL. When IVLBCL is suspected, accessible extracranial tissue, particularly skin, should be considered before proceeding to brain biopsy; cerebral tissue sampling may still be required when extracranial investigations are inconclusive or the intracranial diagnosis remains uncertain.

Neurological manifestations could also occur without detectable MRI lesions. In our cohort, two patients with encephalopathy had normal brain MRI, while one patient with clinically localized myelopathy showed no corresponding spinal cord abnormalities. Similar clinical–imaging discrepancies have been reported: Watanabe et al. described an IVLBCL patient with abnormal behavior and impaired consciousness despite unremarkable brain MRI and cerebrospinal fluid findings [19], while Uawithya et al. reported a patient with conus medullaris syndrome and normal spinal MRI [13]. Microscopic vascular involvement below the detection threshold of conventional imaging is one possible explanation, but this mechanism was not established in our MRI-negative cases. These observations suggest that imaging findings may incompletely reflect neurological dysfunction. In these patients, further diagnostic evaluation should be guided by clinical localization, with electrophysiological testing when peripheral involvement is suspected and assessment for systemic causes when the neurological manifestations remain unexplained.

Peripheral involvement in our cohort presented as bilateral lumbosacral radiculoneuropathy with lower-limb weakness, without a sensory level or other clear signs localizing to the spinal cord. Unlike some previous reports, we did not observe an asymmetric mononeuropathy multiplex presentation [20]. Given the small numbers in both our cohort and previous reports, this difference may reflect the heterogeneous distribution of peripheral involvement in IVLBCL. Nerve-root thickening and enhancement can also occur in inflammatory polyradiculoneuropathies [21]. Without nerve-root histopathology, we could not determine whether the abnormalities reflected microvascular occlusion with ischemic injury, direct lymphomatous infiltration, or associated inflammation.

Neurologically asymptomatic involvement was identified in eight patients. Two of the four patients with brain MRI abnormalities had solitary lesions, suggesting that limited lesion burden may partly account for the absence of neurological manifestations. The other four patients had pituitary hypermetabolism, including two with pituitary-related endocrine abnormalities. Previous reports have documented IVLBCL-related pituitary involvement without pituitary enlargement or discernible lesions on MRI [22,23]. Endocrine dysfunction may therefore occur without overt neurological manifestations related to sellar mass effect. In our patients, pituitary abnormalities were considered likely related to IVLBCL in the context of systemic disease and the absence of a more plausible alternative explanation, but this attribution remained presumptive without histopathological confirmation. Longitudinal assessment of MRI and PET/CT findings after treatment, together with subsequent neurological events, may help clarify the significance of these asymptomatic abnormalities.

Several limitations should be acknowledged. First, this single-center retrospective study had a small sample size, and some imaging studies performed at other hospitals could not be retrieved for review. Spinal cord and nerve-root MRI was performed selectively rather than routinely, limiting assessment of the frequency of imaging abnormalities and precluding estimates of MRI sensitivity. These limitations may introduce bias and restrict generalizability. Second, IVLBCL was diagnosed predominantly through extracranial tissue biopsies, particularly skin biopsy; limited CNS tissue sampling prevented systematic validation of the proposed imaging–pathology relationships. Third, the patterns were derived retrospectively as descriptive categories, not as a validated diagnostic classification. The absence of a neurological mimic control group, external validation, and formal inter-rater agreement assessment limits conclusions regarding their diagnostic performance and reproducibility. Finally, the small number of patients evaluable for treatment outcomes precluded treatment subgroup analyses.

In conclusion, neurological involvement in IVLBCL encompasses diverse clinical and imaging manifestations that may coexist, evolve over time, or occur without corresponding abnormalities on clinical examination or MRI. The six clinicoradiologic patterns described here offer a structured approach to recognizing these presentations and considering IVLBCL when an initial diagnosis does not adequately explain the clinical course or imaging findings. Assessment should integrate neurological localization with systemic findings, including in patients without overt neurological symptoms. Future studies should examine neurological recovery across these patterns to better characterize the functional consequences of the disease and its treatment.

## Figures and Tables

**Table 1:** Clinical characteristics of patients with neurological involvement.

	All Patients (*N* = 29) ^a^
**Age, y** **ears (median, IQR)**	57 (52–64)
**Gender (male, %)**	18 (62.1)
**Ann Arbor stage IV, *n* (%)**	29 (100)
**IPI score (median, range)**	4 (2–5)
**Time to diagnosis ^b^, days (median, IQR)**	145 (68–193)
**Patients with neurologic manifestations, *n* (%)**	21 (72.4)
**No neurologic manifestation, *n* (%)**	8 (27.6)
**Neurologic symptoms and signs, *n* (%)**	
Cognitive decline or slowed responses	10 (34.5)
Seizures	6 (20.7)
Decreased consciousness	7 (24.1)
Focal neurologic deficits ^c^	5 (17.2)
Sphincter disturbance	12 (41.4)
Bilateral lower-limb weakness	9 (31.0)
Sensory level	3 (10.3)
Headache and vomiting	1 (3.4)
**Systemic symptoms, *n* (%)**	
Fever	21 (72.4)
Dyspnea	12 (41.4)
Cytopenia	23 (79.3)
Hepatosplenomegaly	12 (41.4)
Unexplained edema	10 (34.5)
Cutaneous lesions	8 (27.6)
Endocrine abnormalities	3 (10.3)
**Serum markers**	
LDH, U/L [*n* = 29] (median, IQR)	896 (419–1151)
IL-6, pg/mL [*n* = 25] (median, IQR)	31 (8–60)
IL-10, pg/mL [*n* = 25] (median, IQR)	323 (101–1000)
Autoantibodies positive [*n* = 29] (%)	7 (24.1)
**Cerebrospinal fluid markers**	
Cell counts, ×10^6^/L [*n* = 29] (median, IQR)	2 (0–4)
Protein, g/L [*n* = 29] (median, IQR)	0.72 (0.39–1.17)
IL-6, pg/mL [*n* = 21] (median, IQR)	7 (3–103)
IL-10, pg/mL [*n* = 22] (median, IQR)	6 (5–25)
**Biopsy positive site [*N* = 29] ^d^, *n* (%)**	
Skin	17 (58.6)
Lung	3 (10.3)
Bone marrow	3 (10.3)
Lymph node	2 (6.9)
Liver	1 (3.4)
Upper aerodigestive tract ^e^	2 (6.9)
Brain	3 (10.3)

**^a^**. Clinical symptoms and signs were assessed in all 29 patients. Bracketed *n* values indicate the number of patients tested for each laboratory investigation. **^b^**. Time to diagnosis was defined as the interval from the onset of the first symptom considered attributable to IVLBCL, whether neurological or systemic, to histopathological confirmation of IVLBCL. **^c^**. Focal neurological deficits included facial palsy, monoparesis, hemiplegia, hemisensory loss, and aphasia. **^d^**. Biopsy percentages represent the proportion of all 29 patients with a positive biopsy at each site, rather than the diagnostic yield among patients biopsied at that site. **^e^**. The upper aerodigestive tract included the paranasal sinus and posterior pharyngeal wall. **Abbreviations**: IPI, International Prognostic Index; IQR, interquartile range; LDH, lactate dehydrogenase; IL, interleukin.

**Table 2:** MRI and FDG-PET/CT features of patients with neurological involvement.

Characteristics	All Patients (*N* = 29)
**Brain MRI availability [*N* = 29], *n* (%)**	23 (79.3)
No lesions in brain MRI [*n* = 23], *n* (%)	8 (34.8)
Multiple brain lesions [*n* = 23], *n* (%)	13 (56.5)
Single brain lesion [*n* = 23], *n* (%)	2 (8.7)
**Intracranial location [*n* = 23] ^a^, *n* (%)**	
Subcortical white matter	12 (52.2)
Cerebral cortex	4 (17.4)
Brainstem	7 (30.4)
Basal ganglia	4 (17.4)
Corpus callosum	3 (13.0)
Cerebellum	3 (13.0)
**Brain MRI lesion findings [*n* = 23], *n* (%)**	
Infarct-like or ischemic lesions	9 (39.1)
Mass-like lesions with contrast enhancement	1 (4.3)
White matter hyperintensities	10 (43.5)
Hemorrhagic lesions	3 (13.0)
**Other neurological MRI findings, *n* ^b^**	
Spinal cord lesions	2
Nerve-root thickening and/or enhancement	4
**^18^ F-FDG-PET/CT features [*n* = 22] ^c^, *n* (%)**	
Hypermetabolism in neural structures or the pituitary gland	11 (50.0)
Cerebral or meningeal hypermetabolism	4 (18.2)
Nerve roots	1 (4.5)
Pituitary gland	7 (31.8)
Systemic hypermetabolism	17 (77.3)
**Correspondence between brain MRI and FDG-PET/CT findings [*n* = 16] ^d^, *n* (%)**	
Hypermetabolism corresponding to cerebral MRI T2-hyperintense lesions	2 (12.5)
Hypometabolism in cerebral MRI ischemic lesions	1 (6.3)
Hypermetabolism corresponding to enhancing mass-like lesions	1 (6.3)
No abnormal FDG uptake corresponding to MRI lesions	6 (37.5)
No brain MRI lesions or abnormal FDG uptake in neural structures or the pituitary gland	4 (25.0)
Nerve-root or pituitary hypermetabolism without MRI lesion	2 (12.5)

**^a^**. Brain MRI was available for 23 of the 29 patients. **^b^**. Spinal MRI and lumbosacral nerve-root MRI were performed selectively based on clinical indications rather than routinely in all patients; therefore, spinal cord and nerve root lesions are reported as absolute numbers only. **^c^**. FDG-PET/CT was available for 22 patients. **^d^**. PET/CT and Cranial MRI were both available for 16 patients.

## Data Availability

The datasets generated and analyzed during the current study are not publicly available to protect patient privacy but are available from the corresponding author on reasonable request, subject to applicable privacy and ethical restrictions.
